# Phenotype of NK-Like CD8(+) T Cells with Innate Features in Humans and Their Relevance in Cancer Diseases

**DOI:** 10.3389/fimmu.2017.00316

**Published:** 2017-03-27

**Authors:** Alice Barbarin, Emilie Cayssials, Florence Jacomet, Nicolas Gonzalo Nunez, Sara Basbous, Lucie Lefèvre, Myriam Abdallah, Nathalie Piccirilli, Benjamin Morin, Vincent Lavoue, Véronique Catros, Eliane Piaggio, André Herbelin, Jean-Marc Gombert

**Affiliations:** ^1^INSERM 1082, Poitiers, France; ^2^CHU de Poitiers, Poitiers, France; ^3^Service d’Hématologie et d’Oncologie Biologique, CHU de Poitiers, Poitiers, France; ^4^Université de Poitiers, Poitiers, France; ^5^Service d’Immunologie et Inflammation, CHU de Poitiers, Poitiers, France; ^6^Institut Curie, PSL Research University, INSERM U932, Paris, France; ^7^SiRIC Translational Immunotherapy Team, Translational Research Department, Research Center, Institut Curie, PSL Research University, Paris, France; ^8^Centre d’Investigation Clinique Biothérapie CICBT 1428, Institut Curie, Paris, France; ^9^INSERM U1242, Rennes, France; ^10^CHU de Rennes, Rennes, France; ^11^INSERM U991, Rennes, France; ^12^CRB Santé de Rennes, Rennes, France

**Keywords:** innate memory CD8(+) T cells, NK-like T cells, iNKT cells, natural killer receptors, Eomesodermin, CD49d, chronic myeloid leukemia, solid cancers

## Abstract

Unconventional T cells are defined by their capacity to respond to signals other than the well-known complex of peptides and major histocompatibility complex proteins. Among the burgeoning family of unconventional T cells, innate-like CD8(+) T cells in the mouse were discovered in the early 2000s. This subset of CD8(+) T cells bears a memory phenotype without having encountered a foreign antigen and can respond to innate-like IL-12 + IL-18 stimulation. Although the concept of innate memory CD8(+) T cells is now well established in mice, whether an equivalent memory NK-like T-cell population exists in humans remains under debate. We recently reported that CD8(+) T cells responding to innate-like IL-12 + IL-18 stimulation and co-expressing the transcription factor Eomesodermin (Eomes) and KIR/NKG2A membrane receptors with a memory/EMRA phenotype may represent a new, functionally distinct innate T cell subset in humans. In this review, after a summary on the known innate CD8(+) T-cell features in the mouse, we propose Eomes together with KIR/NKG2A and CD49d as a signature to standardize the identification of this innate CD8(+) T-cell subset in humans. Next, we discuss IL-4 and IL-15 involvement in the generation of innate CD8(+) T cells and particularly its possible dependency on the promyelocytic leukemia zinc-finger factor expressing iNKT cells, an innate T cell subset well documented for its susceptibility to tumor immune subversion. After that, focusing on cancer diseases, we provide new insights into the potential role of these innate CD8(+) T cells in a physiopathological context in humans. Based on empirical data obtained in cases of chronic myeloid leukemia, a myeloproliferative syndrome controlled by the immune system, and in solid tumors, we observe both the possible contribution of innate CD8(+) T cells to cancer disease control and their susceptibility to tumor immune subversion. Finally, we note that during tumor progression, innate CD8(+) T lymphocytes could be controlled by immune checkpoints. This study significantly contributes to understanding of the role of NK-like CD8(+) T cells and raises the question of the possible involvement of an iNKT/innate CD8(+) T cell axis in cancer.

## Introduction

The traditional view of the immune system distinguishes innate immunity from adaptive or acquired immunity. Innate immunity is derived from cells expressing the receptors specific for molecules from microbial pathogens called pathogen-associated molecular patterns or self-molecules from the healthy or unhealthy individual called damage-associated molecular patterns. One of the key characteristics of innate immunity effector mechanisms is their capacity for very rapid response to pro-inflammatory cytokines such as IL-12, IL-18, and IL-33.

The effectors of adaptive immunity possess receptors characterized by highly diverse and specific antigens. A major feature of adaptive immunity consists in its serving as an essential support for the immunologic memory, which means that it can remember and quite effectively respond to an antigen long after having encountered it for the first time.

However, numerous works over the past 20 years have shown the distinction between innate immunity and adaptive immunity to be less clear-cut and more tenuous than it first appeared. This revised perception is based, in particular, on description of non-conventional T cells responding to *stimuli* that had previously been considered as being recognized solely by innate cells. These populations of T lymphocytes include not only certain T-cell receptor (TCR)-γδ cells but also TCR-αβ cells such as natural killer T (iNKT) cells and innate mucosal-associated invariant T (MAIT) cells [for a list of the different cells, see Ref. (1, 2)].

A new contingent of innate T cells was described in the early 2000s in the mouse, partially in the thymus, where they were termed «innate memory» (IM) CD8(+) T cells, and partially in the spleen, where they were termed «virtual memory» (VM) CD8(+) T cells (3, 4). Aside from possessing a phenotype of activated memory cells, one characteristic of these cells consists in their differentiating into memory cells independently of a foreign antigen. In parallel, CD8(+) T cells in humans were described as cells possessing innate characteristics including NK markers. They were found in human cord blood, a finding consistent with the hypothesis that their development does not depend on foreign antigens. These cells hence were termed NK-like CD8(+) T cells.

At the outset of this review, we shall compare the human NK-like CD8(+) T cells with IM/VM CD8(+) T cells in mice. On the basis of this comparison and with regard to humans, we shall focus first on expression of the transcription factor Eomesodermin (Eomes) as a lineage marker of that population of cells, and then on their innate functions (cytotoxicity and TCR-independent IFN-γ expression), along with their memory phenotype, and on the roles of IL-4- and promyelocytic leukemia zinc-finger factor (PLZF)-expressing T cells in differentiation of these cells, hereafter referred to as innate CD8(+) T cells. We shall discuss the use of membrane markers, particularly the α4-integrin CD49d, in order to obtain a more well-defined phenotype correlating with their functions and/or explaining their possible physiological role. Finally, we shall discuss the implication of innate CD8(+) T cells in anticancer immunity in humans.

## Innate CD8(+) T Lymphocytes in Mice

Studies conducted shortly after 2000 by Forman et al. (5, 6) demonstrated the existence of CD8(+) T cells producing IFN-γ in response to innate signals occurring independently from the TCR. These CD8(+) T cells possessed a CD44(+) CD62L(−) CD122(+) memory phenotype and, *in vivo*, provided protection against *Listeria monocytogenes* (LM) infection (5–7). Their mobilization depended on the production of IL-12 and IL-18. Interestingly, the Forman team subsequently showed that this cell population was present in the thymus of C57BL/6 wild-type mice and that it was enriched in C57BL/6 H-2 K^b−/−^D^b−/−^ mice not having undergone stimulation by foreign antigens (7).

A second series of studies having to do with Itk^−/−^ (inducing T cell kinase), Rlk^−/−^ (resting lymphocyte kinase), or Itk^−/−^Rlk^−/−^ mice led to identification of a population of thymic CD8(+) T cells expressing an activated memory [CD44(+) CD62L(−) CD122(+)] phenotype and called IM CD8(+) T cells (8–12). Interestingly, these cells developed in the thymus and are exported to the spleen and the lymph nodes (LNs) where they could fulfill an anti-infective function against LM, particularly through production of IFN-γ following TCR-independent stimulation by IL-12 and IL-18. An important point in the studies dedicated to this population has been the demonstration that its differentiation depended on expression of the Eomes transcription factor and IL-15 (13–15). Eomes expression initiates the differentiation program of these cells and induces the expression of CD122 (the β chain of the IL-2 and IL-15 receptor). IL-15 has a critical role in the expansion of IM CD8(+) T cells (8, 16). It should also be noted that this population is present in Itk^−/−^K^b−/−^D^b−/−^ mice, a finding suggesting that at least some IM CD8(+) T cells are selected by non-classical major histocompatibility complex (MHC) class I molecules (9, 17–19).

A final series of studies has described in mouse spleens and peripheral lymphoid organs a population of activated [CD122(+)] and memory [CD44(+) CD62L(−)] CD8(+) T lymphocytes of which the differentiation into memory cells occurs independently of any recognition of a foreign antigen. This population consists in the so-called VM CD8(+) T cells. As is the case with IM T CD8(+) cells, Eomes and IL-15 are the two key factors in their differentiation. This population is capable of producing IFN-γ in response to innate stimulation by IL-12 + IL-18 (11, 20–22).

**Figure 1:**
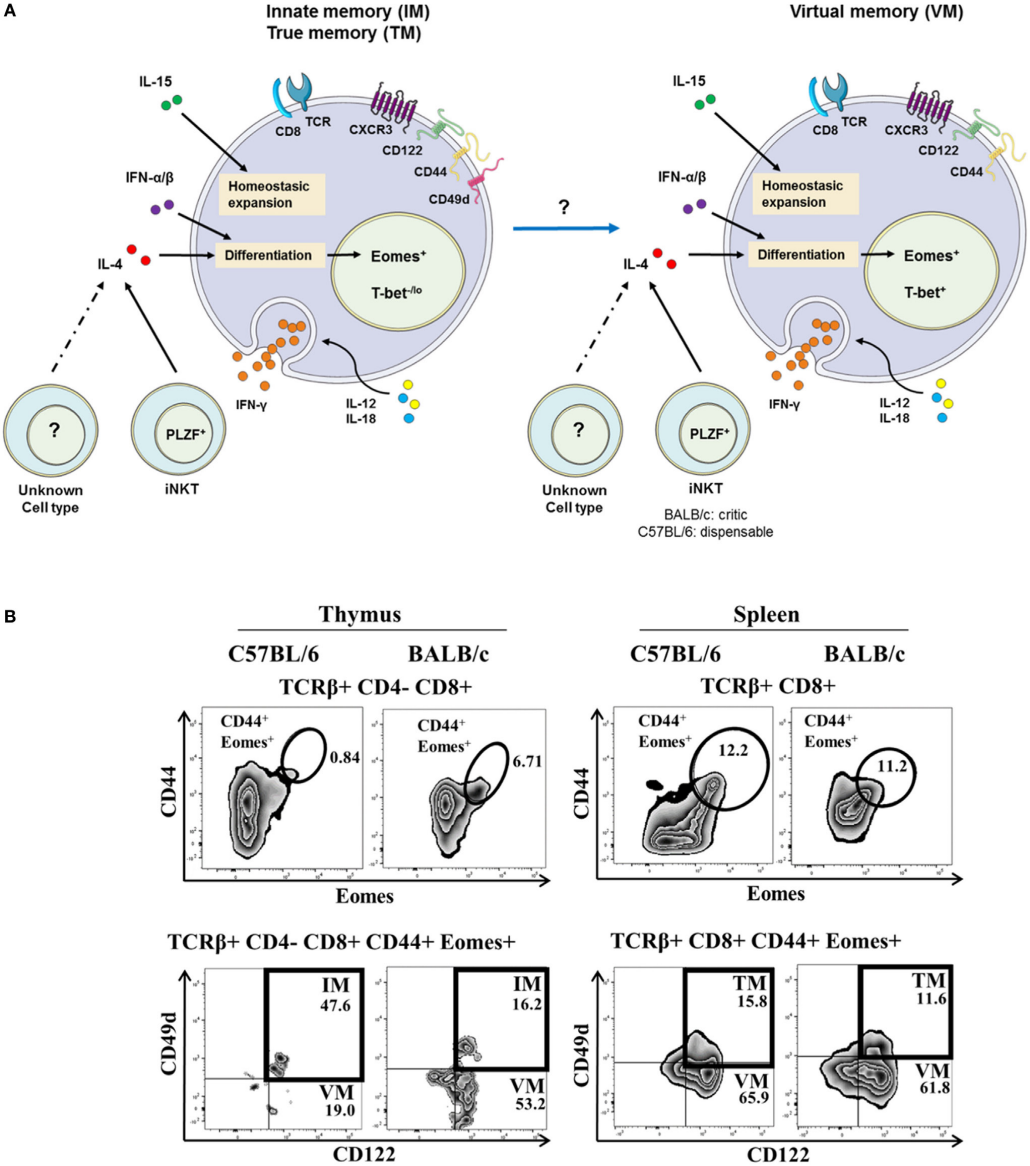
**The innate/true memory (IM/TM) and virtual memory (VM) CD8(+) T cells in mice**. **(A)** Representative view of IM/TM and VM CD8(+) T cells in mice. CXCR3(+) CD122(+) CD44(+) CD49d(+) Eomes(+) and CXCR3(+) CD122(+) CD44(+) Eomes(+) T-bet(+) expression defined the IM/TM CD8(+) and the VM CD8(+) T-cell subsets, respectively. Both subsets have the capacity to respond to the IL-12 + IL-18 innate-like stimulation, and their development and homeostasis are regulated by IL-4 [mainly produced by PLZF(+) iNKT cells], IL-15, and type I interferons. Note that possible existence of a filiation link in mice between IM CD8(+) and VM CD8(+) T-cell populations remains to be investigated. **(B)** Gating strategy in C57BL/6 and BALB/c mouse strains for IM and VM CD8(+) T cells in the thymus (left panel) and for TM and VM CD8(+) T cells in the spleen (right panel). IM/TM and VM population frequencies are displayed in the upper and lower right quadrants, respectively.

The expression by mouse thymic IM CD8(+) T cells of some integrins, such as CD49d [an α4-integrin, or VLA-α4, which is most often matched with a β7-integrin (which is bound to Madcam and VCAM-1 or CD106), or a β1-integrin], has been described (14, 21, 23, 24). Hence, CD49d is used to discriminate between IM and VM T cells (Figure 1A) arising from the thymus or the spleen, respectively. In this model, a possible filiation link between IM CD8(+) and VM CD8(+) T-cell populations remains to be investigated. A gating strategy to identify IM/VM CD8(+) T cells in mice, taking Eomes, CD44, and CD122 together with CD49d as delineating markers is depicted in Figure 1B and Figure S1 in Supplementary Material. Based on this gating strategy, in the C57BL/6 mouse strain, the vast majority of memory [CD44(+) Eomes(+) CD122(+)] CD8(+) T cells are IM CD8(+) T cells in the thymus vs. VM CD8(+) T cells in the spleen, as attested by their differential CD49d expression. Interestingly, in the BALB/c strain, thymic IM CD8(+) T cells are minority cells among memory CD44(+) CD122(+) Eomes(+) cells, raising the question of a possible link between IM and VM CD8(+) T cells, as well as the association of CD49d with the innate functions of IM/VM CD8(+) T cells (see CD49d, a Functional Marker of Innate CD8(+) T Cells in Humans).

IL-15 plays a key role in the homeostatic expansion of CD8(+) T cells, and it has been reported in several studies that this cytokine is implicated in differentiation into VM CD8(+) T cells after homeostatic proliferation (4, 25–28). However, August and his team have shown that the size of the IM/VM CD8(+) T population is not modified by T-cell depletion prior to bone marrow transplantation, a finding suggesting that lymphoid precursors are differentiated into IM/VM CD8(+) T cells following “tuning” by cells expressing MHC class I molecules. Moreover, in the same study, IM/VM CD8(+) T cells are distinguished from homeostatic proliferation CD8(+) T cells by a different transcriptional profile (29). Another study suggests that contrary to naive CD8(+) T cells and homeostatic proliferation CD8(+) T cells, acquisition of the phenotype of IM CD8(+) T cells necessitates engagement of their TCR (30).

## Evidence for NK-Like CD8(+) T Cells in Humans

In parallel with the previously described work on mice, several studies conducted in the early 2000s demonstrated the existence in humans of CD8(+) T cell-expressing markers and receptors of NK cells including CD56, KIR, NKG2A and NKG2C (CD159a and c), and CD94 (31–36). Several studies precisely characterized the phenotype of the KIR(+) CD8(+) T cells and showed that they possess an EMRA memory phenotype [CD45RA(+) CCR7(−) CD57(+)] (33, 34). Finally, Björkström et al. (33), showed that EMRA(+) KIR(+) CD8(+) T cells have a skewed repertoire using fewer different Vβ than their EMRA(+) KIR(−) CD8(+) T cell counterpart, a finding suggesting the role of antigenic pressure in the acquisition of this phenotype. An equivalent to this population of KIR/NKG2(+) CD8(+) T cells is present in human cord blood, where they possess an EMRA memory phenotype and rapidly express IFN-γ following TCR stimulation (36). In fact, this is a population of T cells that has been educated and has differentiated in the absence of foreign antigenic stimulation, into terminal effector memory T cells.

These KIR(+) CD8(+) T cells have a weaker response to TCR stimulation than their KIR(−) CD8(+) counterparts with regard to the expression of IFN-γ and TNF-α or to the degranulation evaluated by CD107a staining. The KIR(+) CD8(+) T cells expressing two different KIRs have a weaker response to TCR stimulation than cells expressing a single KIR or without KIR (33, 34). Remarkably, this CD56(+) [or KIR(+)] CD8(+) T cell subset responds quite effectively to innate *stimuli*, one example being the association of IL-12 with IL-18 (37). The same team reported that loss of this function in *IL-12R^−/−^* patients is associated with a risk of severe infections from intracellular germs, particularly mycobacteria and *Salmonella* (37).

## Human NK-Like CD8(+) T Cells Express Eomes and Display Innate Functions

**Figure 2:**
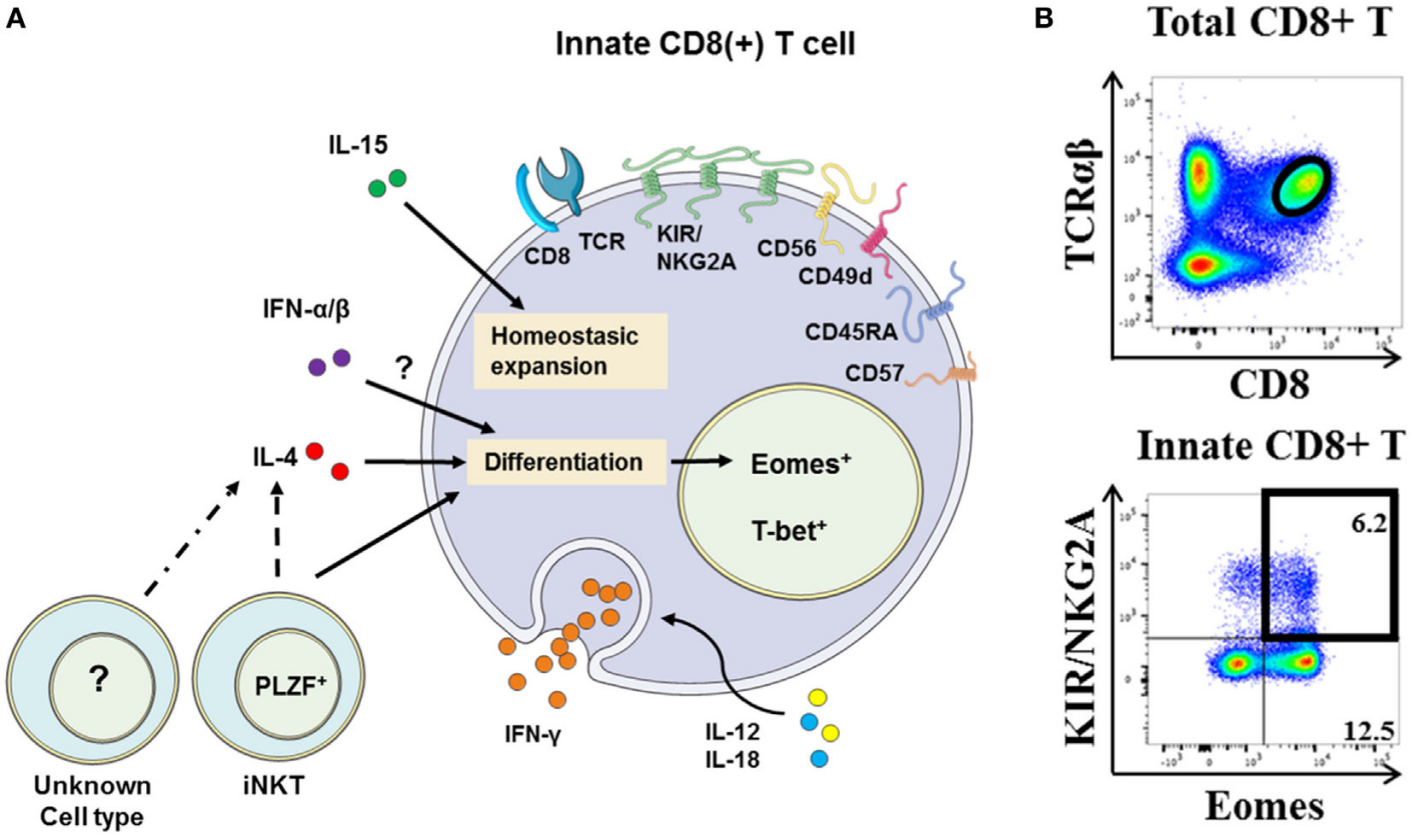
**The innate CD8(+) T cells in humans**. **(A)** Representative view of human innate CD8(+) T cells. The membrane markers KIR/NKG2A, CD56, CD57, CD45RA, and CD49d and nuclear transcription factors Eomes and T-bet define this new unique innate CD8(+) T-cell population [for details, see Ref. (4, 38)]. Innate CD8(+) T cells in humans have the capacity to respond to IL-12 + IL-18 innate-like stimulation. Their development and homeostasis are regulated by IL-15, IL-4 [presumably by PLZF(+) iNKT cells], and expectedly type I interferons. Note that the precise origin (central vs. peripheral) of innate CD8(+) T-cell populations in humans remains under debate. **(B)** Gating strategy for innate CD8(+) T cells among peripheral blood mononuclear cells from healthy donors. After gating on T-cell receptor (TCR)-αβ(+) CD8(+) cells, the innate CD8(+) T cells are defined as KIR/NKG2A(+) Eomes(+). Frequency of innate CD8(+) T cells is displayed in the upper right corner.

**Figure 3:**
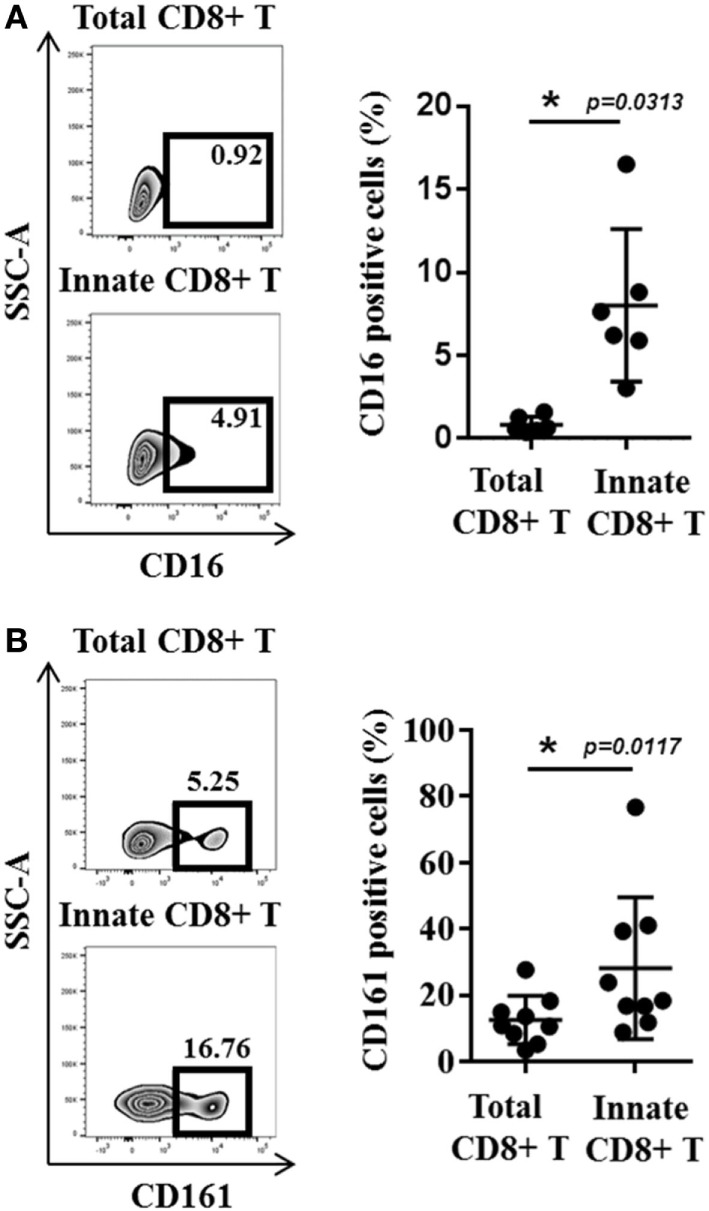
**CD16 and CD161 are slightly enriched in innate CD8(+) T cells**. **(A)** Peripheral blood mononuclear cells (PBMCs) from healthy donors (HDs) (*n* = 6) were cultured for 48 h in the presence of IL-15 and then analyzed by flow cytometry. One representative sample is shown for CD16 expression among total (upper panel) and innate (lower panel) CD8(+) T-cell subsets. Frequencies are displayed in the gate and the histogram (right panel) presents the full cohort of CD16-positive cells (mean ± SD) in both T-cell subsets. **(B)** PBMCs from HD (*n* = 9) were analyzed *ex vivo* by flow cytometry. One representative sample is shown for CD161 expression among total (upper panel) and innate (lower panel) CD8(+) T-cell subsets. Frequencies are displayed in the gates (left panel). The histogram (right panel) represents the full cohort of CD161-positive cells (mean ± SD) in both T-cell subsets.

The study by Jacomet et al. (38) showed that KIR(+) and/or NKG2A(+) CD8(+) T cells preferentially express Eomes. These KIR/NKG2A(+) Eomes(+) CD8(+) T cells have a memory phenotype and share functional and phenotypic features (4, 38) with IM CD8(+) T cells in mice (8–11). Moreover, with respect to KIR/NKG2A(−) CD8(+) T cells, they possess an EMRA phenotype [CD45RA(+) CCR7(−)] and preferentially express the surface molecule CD57, which is a terminal differentiation marker (Figure 2A). Figure 2B shows a gating strategy designed to analyze these cells in human blood. Remarkably, the majority (around 60–70%) of KIR/NKG2A(+) Eomes(+) CD8(+) T cells express the T-box transcription factor T-bet, a phenotype comparable to the phenotype described for mouse VM CD8(+) T cells (22). These cells are characterized by increased frequency of CD16(+) cells in comparison to conventional memory [KIR/NKG2A(−) Eomes(+)] CD8(+) T cells. Moreover, CD16 expression is substantially increased in KIR/NKG2A(+) Eomes(+) CD8(+) T cells following 48 h of IL-15 *in vitro* treatment (Figure 3A).

Together with their marked NK-like phenotype, a hallmark of KIR/NKG2A(+) Eomes(+) CD8(+) T cells is their capacity to rapidly produce large amounts of IFN-γ in response to innate-like stimulation by IL-12 + IL-18 (38). So it is that among the CD8(+) T cells, 60–70% of those possessing a capacity for innate response to IL-12 + IL-18 are KIR/NKG2A(+) Eomes(+) CD8(+) T cells. Moreover, the frequency of the cells producing IFN-γ in response to IL-12 + IL-18 stimulation is four times greater than that of the same cells stimulated with anti-CD3 and anti-CD28 agonistic monoclonal antibodies (mAbs) (38). In the same way, these cells possess a cytotoxic arsenal: perforin and granzyme B. They possess an innate cytotoxic potential induced by an anti-CD16 antibody and revealed by the CD107a test, which assesses degranulation (38). In the final analysis, these data demonstrate that KIR/NKG2A(+) Eomes(+) CD8(+) T cells display innate activity by responding to innate *stimuli* with response efficacy greater than that of their response to adaptive *stimuli* (*i.e*., *via* their TCR), as has also been shown for innate T-cell populations such as dendritic epidermal T cells (39).

A final element appearing to favor the innate character of KIR/NKG2A(+) Eomes(+) CD8(+) T cells is their differentiation without any exogenous antigenic stimulation, as it is possible to show the existence of these cells in the fetal thymus (40), and as our team has shown them to be present in cord blood. Interestingly, in cord blood, they express an EMRA phenotype with a lower CD57 cell frequency than in the adult, suggesting that there exist supplementary steps in the terminal maturation of these peripheral cells (38). Functionally, as is the case in adult cells, these KIR/NKG2A(+) Eomes(+) CD8(+) T cord blood cells express IFN-γ after innate-like stimulation by IL-12 + IL-18.

All in all, we described for the first time a CD8(+) T cells population with innate features by associating Eomes and KIR/NKG2A markers with the capacity to respond to IL-12 + IL-18 stimulation. These features validate the KIR/NKG2A(+) Eomes(+) CD8(+) T-cell compartment as a new member of the innate T cell family in humans, and we have termed them innate CD8(+) T cells (41).

There exist a number of hypotheses on the mechanisms involved in the reprogramming of conventional T lymphocytes into innate CD8(+) T lymphocytes. One of these assumptions is based on a cross talk between IL-12R and TCR signalosome, in which IL-12 recruits Tyk2 and Fyn tyrosine kinases to activate CD3ζ–TCR signal transduction pathways (42).

Some studies conducted in mice suggest that IM and/or VM CD8(+) T cells could be selected by non-classical MHC class I molecules (9, 17–19). In humans, our results (Figure S2 in Supplementary Material) show that a relatively small fraction of KIR/NKG2A(+) Eomes(+) CD8(+) cells are MAIT cells. This finding suggests that at least some human innate CD8(+) T cells could be selected by non-classical class I MHC molecules, and it raises the possibility of the presence in this population of cells being restricted by non-classical MHC class I HLA-E molecules (18).

## CD49d, a Functional Marker of Innate CD8(+) T Cells in Humans

We have sought out other markers of innate CD8(+) T cells in humans. Among them, we tested CD56, a marker in humans associated with NK cells, but our results showed that this marker is no more effective in distinguishing the innate CD8(+) T-cell population than the KIR/NKG2A markers. More precisely, this marker delineate only 20–30% of the CD8(+) T cells expressing IFN-γ after innate stimulation by IL-12 + IL-18 (as opposed to the approximately 70% exhibited by the KIR/NKG2A markers) [data not shown; (38)]. We also tested CD161, of which the expression is a common feature of human innate T cell subsets including iNKT cells, TCR-γδ T cells, and MAIT cells (43). However, as for CD56, CD161 is expressed by only approximately 20% of the Eomes(+) KIR/NKG2A(+) CD8(+) T cells (Figure 3B).

**Figure 4:**
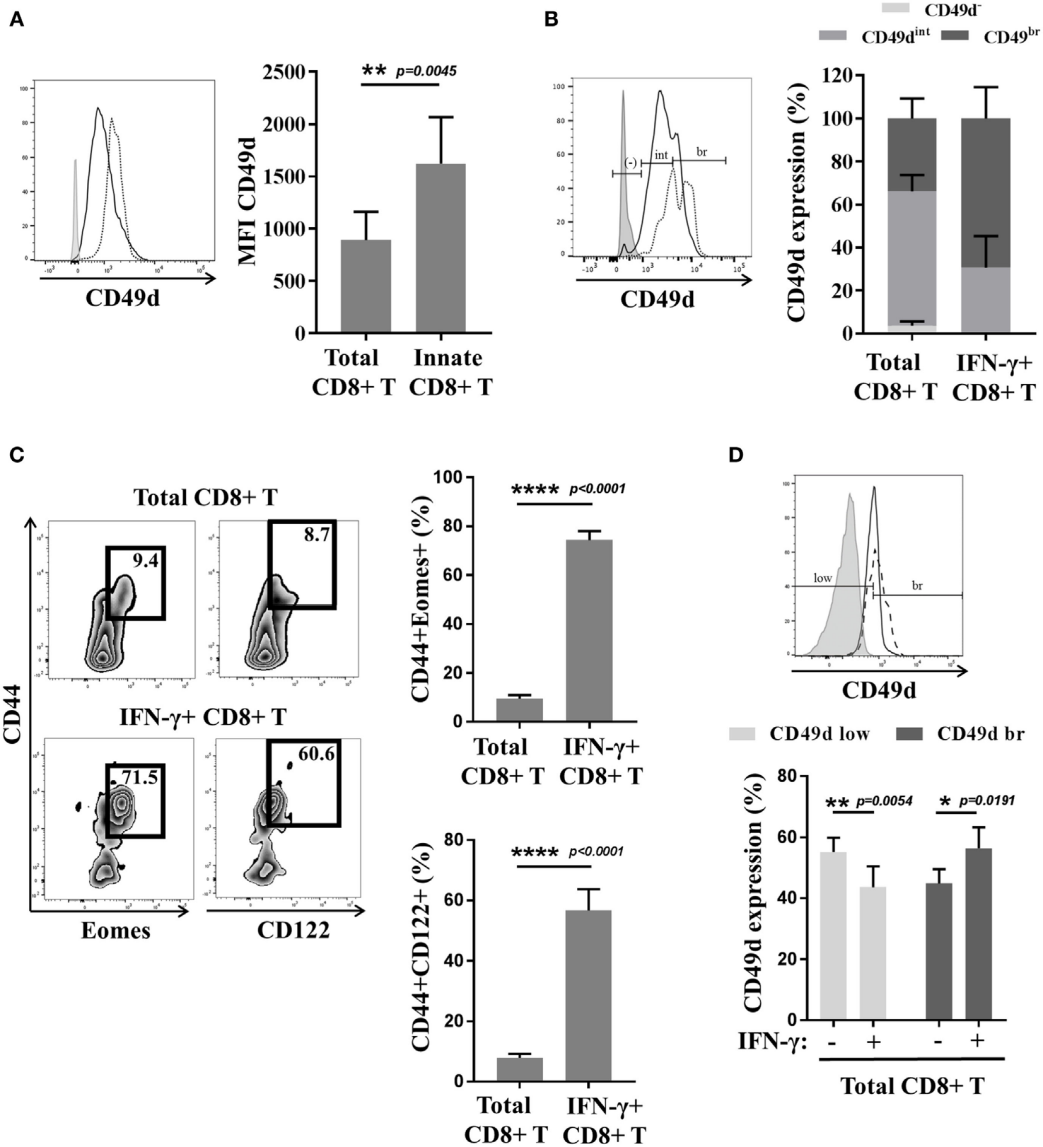
**The CD49d molecule correlates with the innate function of innate CD8(+) T cells in mice and humans**. **(A)** Peripheral blood mononuclear cells (PBMCs) from healthy donors (HDs) (*n* = 6) were analyzed *ex vivo* by flow cytometry for CD49d expression in total and innate CD8(+) T cells. One representative sample is shown (left): total CD8(+) T cells (solid dark line), innate CD8(+) T cells (dotted black line), and isotype control (gray). Full cohort (MFI ± SD) is shown on the histogram (right). **(B)** PBMCs from HD (*n* = *5*) were cultured 48 h in the presence of IL-12 + IL-18 and then analyzed *ex vivo* by flow cytometry for CD49d expression. Distribution of CD49d (percentage ± SD) in total CD8(+) T cells and in IFN-γ(+) CD8(+) T cells is shown on the histogram (right). One representative sample is shown (left): total CD8(+) T cells (solid dark line), IFN-γ(+) CD8(+) T cells (dotted black line), and isotype control (gray). **(C,D)** Splenocytes from Eomes-GFP mice were isolated, cultured for 16 h in the presence of IL-12 + IL-18, and analyzed by flow cytometry. **(C)** One representative sample is shown for the presence of CD44(+) Eomes(+) and CD44(+) CD122(+) cells among total CD8(+) (upper left panel) or IFN-γ(+) CD8(+) T cells (lower left panel). Frequency of each subset is displayed in the gate. Full cohort frequencies for CD44(+) Eomes(+) (upper histogram) and CD44(+) CD122(+) (lower histogram) cells among total CD8(+) and IFN-γ(+) CD8(+) T cells are shown. **(D)** CD49d expression was analyzed in total and IFN-γ(+) CD8(+) T cells. One representative sample is shown (upper panel): total CD8(+) T cells (solid dark line), IFN-γ(+) CD8(+) T cells (dotted black line), and isotype control (gray). Full cohort histograms for frequencies of CD49d(low) and CD49d(bright) are shown (lower panel) in total CD8(+) and IFN-γ(+) CD8(+) T cells.

Reasoning by analogy with the mouse model, we tested CD49d expression by innate CD8(+) T cells in humans (Figures 4A,B). Interestingly, the CD8(+) T cells with the KIR/NKG2A(+) Eomes(+) phenotype strongly expressed CD49d, as compared to the CD8(+) T-cell population taken as a whole (Figure 4A). Moreover, the majority (almost 70%) of the cells with a more pronounced expression of CD49d consisted in those expressing IFN-γ after innate-like stimulation by IL-12 + IL-18 (Figure 4B). These results show CD49d to be closely associated with the innate effector functions of the innate CD8(+) T cells. On the other hand, CD49d cannot substitute for Eomes and KIR/NKG2A, given the fact that only 20–40% of CD49d(+) cells are actually innate CD8(+) T cells (Figure S3 in Supplementary Material). However, as innate CD8(+) T cells arise from the thymus, the functional link between higher CD49d expression and IFN-γ secretion in response to IL-12 + IL-18 stimulation should be tested in cord blood, which is an accessible source of cells providing an approximate reflection of thymic cells in humans.

These results have led us to inquire about the biological meaning of CD49d expression by thymic vs. splenic (or IM vs. VM) CD8(+) T lymphocytes in mice. The results of several teams (14, 21) suggest that contrary to thymic IM CD8(+) cells, splenic (so-called VM) innate CD8(+) T cells do not express CD49d (14, 21) (Figure 1). We have observed, as in humans, that a large majority of splenic CD8(+) T cells expressing IFN-γ after innate-like stimulation by IL-12 + IL-18 were those harboring the IM [Eomes(+) CD44(+) CD122(+)] phenotype (Figure 4C). Moreover, these IFN-γ-producing cells were mostly those that more pronouncedly expressed CD49d as compared to the total CD8(+) T-cell population (Figure 4D).

Taken as a whole, these different results suggest that CD49d is associated with the innate-like functions of NK-like CD8(+) T cells as much in humans as in mice. According to the β chain with which CD49d is matched, the cells are variably liable to migrate toward different territories. While the α4β7-integrin is associated with the migration of T lymphocytes toward the digestive mucosa, the α4β1-integrin is associated with migration toward the oral mucosa, the salivary glands, the vaginal mucosa, and the central nervous system (44, 45). Indeed, therapeutic targeting of CD49d is used in treatment of multiple sclerosis (MS). Several studies have documented the implication of CD49d in the penetration of pathogenic T lymphocytes during MS or experimental allergic encephalomyelitis (EAE) (46, 47). Taken together, these different observations raise the possibility of the implication of the innate CD8(+) T cells in the pathogenesis of MS/EAE.

## The Factors Associated/Implicated in Control of the Differentiation of Innate CD8(+) T Lymphocytes

In mice, differentiation of IM CD8(+) T lymphocytes depends on soluble factors such as type I IFN and the IL-4 and IL-15 cytokines.

Several studies have shown that IL-4 favors the arising of VM CD8(+) T cells (48–51). More recent studies confirm the involvement of IL-4 in IM/VM CD8(+) T cell generation with a more critical role being assumed in the BALB/c (22, 30) than in the C57BL/6 mouse strain (14). IL-4-producing PLZF(+) T cells, including at least partially iNKT lymphocytes (52, 53), elicit the generation of IM CD8(+) T cells. Other results from the Hogquist group confirm the link between iNKT PLZF(+) cells, IL-4, and IM CD8(+) T cell generation by comparing different mouse strains (22). Figure 1B shows the higher frequency of T CD8(+) IM in the thymus of BALB/c in comparison with C57BL/6 mice. However, the August group shows results suggesting that IL-4 produced by differentiating CD4(+) CD8(+) double-positive thymocytes controls the generation of IM CD8(+) thymocytes in the Itk^−/−^ C57BL/6 background (16, 29). As concerns VM CD8(+) T lymphocytes, IL-4 is likewise at least partially implicated. IL-4 is likely to act by eliciting Eomes expression, of which the action would entail a heightened level of expression of CD122, and thereby sensitize the cells to IL-15 (14, 16).

Type I IFNs such as IFN-β and/or IFN-α (24) could favor the differentiation of lymphocytes to VM and/or IM CD8(+) T lymphocytes. The underlying mechanism described by the authors is the induction of Eomes expression by naive CD8(+) T cells.

Finally, IL-15 is a determining factor in the maintenance and/or homeostatic expansion of IM and VM CD8(+) T cells (8, 14, 16).

Few studies have addressed the role of these factors in the development of innate CD8(+) T cells in humans (38, 40). Interestingly, our own results (38) have shown a relation of proportionality between PLZF expression in T and iNKT lymphocytes and Eomes expression by innate CD8(+) T cells in cord blood, suggesting that iNKT cells control the differentiation of human innate CD8(+) T cells. Our results *in vitro* (41) suggest that IL-4 favors Eomes expression and the generation and/or expansion of human innate CD8(+) T cells.

## Is There a Role for Innate CD8(+) T Lymphocytes in Cancer Immunosurveillance?

There exist only sparse data in either humans or mice describing the functions of innate CD8(+) T lymphocytes. An initial set of results consisted in a demonstration in mice of a protective role of IM/VM CD8(+) T lymphocytes against viral (30, 54) and bacterial (11) infections. Our studies have been focused on the numeric/functional status of innate CD8(+) T lymphocytes during the multistep development of human tumors.

### Innate CD8(+) T Lymphocytes in Chronic Myeloid Leukemia (CML)

Similarly to iNKT cells (55–58), innate CD8(+) T cells fulfill functions providing them with anticancer potential. Hence, a deficiency of these cells in CML (Box 1) on diagnosis is likely to constitute, as with iNKT cells (Box 2), an immune subversion signature. If this is indeed the case, parallel study of iNKT cells and innate CD8(+) T cells in CML both at diagnosis and following molecular remission by tyrosine kinase inhibitor (TKI) therapy could perhaps answer questions concerning a dynamic process of generation of innate CD8(+) T cells in humans that would depend on iNKT cells.

Box 1 **Chronic myeloid leukemia (CML), a myeloproliferative syndrome controlled by the immune system.**

Chronic myeloid leukemia is the first malignant disorder with a specific genetic abnormality in the background. It is due to the formation of the chimeric oncogene BCR–ABL. This oncogene is responsible for the transformation of hematopoietic stem cells (HSC) into leukemic stem cells, which results in a leukemic syndrome of mature myeloid cells characterizing the chronic phase (CP) and ineluctably evolving, without treatment, to acute leukemia (59). The ABL domain of the chimeric oncogene BCR–ABL presents deregulated tyrosine kinase activity, which is responsible for the transformation of the HSC. Since the outset of the 2000s, new CML treatment has consisted in the tyrosine kinase inhibitors of BCR–ABL. Some arguments suggest that CML is a disease in which the immune system has a key role [for review, see Ref. (60)]. In addition, during the chronic phase of CML, numerous innate anomalies in the innate immune system have been evidenced. Indeed, defective differentiation of the plasmacytoid dendritic cells, defective IFN-α production by mononuclear cells, and defective functions of NK cells have been observed (61, 62).

Box 2 **Functional deficiency of iNKT cells in chronic phase of CML (CML-CP) patients.**

Our team has shown in CML-CP patients at diagnosis a major defect in the iNKT lymphocyte functions, particularly as concerns their proliferative response to T-cell receptor (TCR) stimulation (63) and their cytotoxic arsenal, with a loss in the expression of perforin and FasL, two elements implicated in cancer immunosurveillance by iNKT cells in mice (57, 64). It must also be emphasized that the iNKT cells of CML-CP patients have lost their expression of the transcription factor promyelocytic leukemia zinc-finger factor and no longer produce IL-4 during TCR engagement; on the other hand, they show normal expression of IFN-γ in comparison with the iNKT lymphocytes of healthy donors or patients in complete remission following treatment by tyrosine kinase inhibitors such as imatinib mesylate (63).

There exists a major defect in the innate CD8(+) T cells of chronic phase of CML (CML-CP) patients compared to those of healthy subjects or patients in complete remission following TKI treatment (41). This numerical defect is associated with a loss of IFN-γ expression after innate-like stimulation by IL-12 + IL-18 cytokines and with a loss of degranulation after stimulation *via* CD16. On the contrary, IFN-γ expression after TCR stimulation (instead of IL-12 + IL-18 stimulation) by the same innate CD8(+) T cells during CP is conserved, thereby showing that the functional defect affecting our population of interest is innate rather than adaptive. Finally, there exists a partial reconstitution in CML remission patients of the frequency and functions of innate CD8(+) T lymphocytes in terms of IFN-γ expression in response to IL-12 + IL-18 and as regards the displaying of cytotoxic functions after CD16 stimulation.

In CML patients, the numerical and functional status of innate CD8(+) T cells seems closely linked to that of the iNKT cells. In analysis of a cohort of CP patients and those in complete remission, we have observed a correlation between Eomes expression by innate CD8(+) T cells and PLZF expression by iNKT cells (41). This finding underscores the possible pathophysiological significance of IL-4 expression by iNKT cells in CML patients; while deficient during the CP, IL-4 expression is restored in remission (63) and could determine the status of innate CD8(+) T cells during the disease or its treatment.

**Figure 5:**
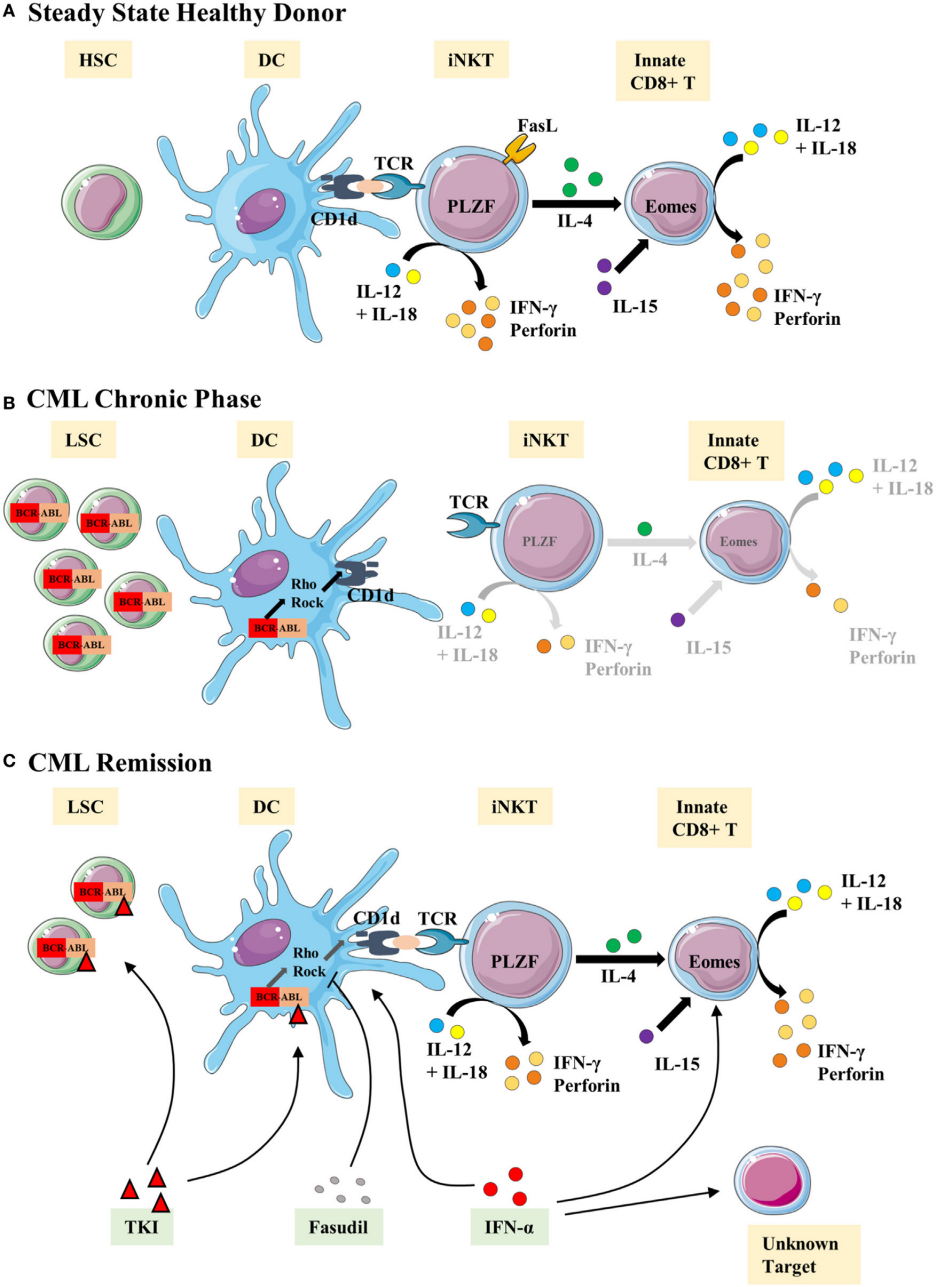
**Representative view of the iNKT/innate CD8(+) T-cell axis hypothesis in chronic myeloid leukemia (CML)**. We propose the following scenario in CML: **(A)** steady state/healthy situation. Normal hematopoietic stem cells (HSC) generate normal immune cells. Antigens are presented *via* the CD1d molecule by dendritic cells (DCs) to iNKT cells. We propose that activated iNKT cells produce IL-4 but the possibility of a T-cell receptor (TCR)-independent mechanism for IL-4 secretion cannot be ruled out. IL-4 is thought to take part with IL-15 in the development/homeostasis of innate CD8(+) T cells. iNKT and innate CD8(+) T cells produce IFN-γ and perforin in response to the innate-like IL-12 + IL-18 stimulation. **(B)** Chronic phase of CML. Leukemic stem cells (LSC) produce modified immune cells bearing BCR–ABL translocation, including DCs. Impaired CD1d antigen presentation by DCs results from activation of the Rho/Rock pathway *via* the DH-PH domain of the ABL part of BCR–ABL. iNKT cell development/stimulation is thereby impaired, especially in terms of promyelocytic leukemia zinc-finger factor (PLZF) expression and IL-4 production. Consequently, we surmise that the innate CD8(+) T subset is defective in number and function. **(C)** Restoration of the iNKT/innate CD8(+) T-cell axis by therapies. IFN-α therapy is thought to help restoring DCs and innate CD8(+) T cells as well as other unidentified cells. Tyrosine kinase inhibitor (TKI) therapies targeting the ABL tyrosine kinase domain clear/control the generation of LSC and abnormal immune cells, including DCs. Fasudil therapy, combined with TKI, restores the CD1d presentation by DCs to iNKT cells and is one possible mechanism to restore the iNKT/innate CD8(+) T-cell axis.

More generally, a scenario can now be outlined as a possible explanation, during CML, for immune subversion of innate CD8(+) T cells by leukemic cells. Immune subversion could result from dysfunction of the antigen-presenting cells (APCs), particularly leukemia myeloid dendritic cells (DCs) and their environment; they might be considered as responsible for the loss of function of the innate immune cells, including NK and innate CD8(+) T cells. As regards iNKT cells, their loss of function could be due to a loss of CD1d expression by the leukemic APCs expressing the BCR–ABL oncogene [Figure 5; (65)]. The loss of CD1d expression could reprogram iNKT cells by favoring the loss of PLZF expression and, consequently, of IL-4, thereby decreasing their capacity to orient a differentiation of CD8(+) T cells into innate CD8(+) T cells (Figure 5).

Another, non-exclusive hypothesis is that of a loss of IL-15 expression by leukemic cells or of the leukemic milieu, as has been shown in colon cancer (66). A different study leads to the suggestion that during CML, TKI might favor the capacity of the DCs to trans-present IL-15 to T cells/NK cells (67). The scope of these works should be broadened, leading to a search for a defect in the expression and trans-presentation of IL-15 during CML-CP. Another relevant element is the loss in sensitivity to IL-15 or signalization of the latter by the innate CD8(+) T cells. Our preliminary results suggest a loss in response to IL-15 by total and innate CD8(+) T lymphocytes (Figure S4 in Supplementary Material). The possible implication of this loss of sensitivity to IL-15 in the dysregulation of the innate CD8(+) T lymphocytes during CML-CP remains to be investigated.

To conclude, CML can be considered as a model in study of the loss of innate effectors (NK cells) and innate T effectors [iNKT cells and innate CD8(+) T cells] and their relevance in the leukemic process. The escape of leukemic cells from the immune system could depend on loss of the coordinated functions of the effectors of classical innate immunity and innate T-cell immunity.

### Innate CD8(+) T Lymphocytes and Solid Tumors

Since we cannot rule out the possibility that the anticancer role attributed to innate CD8(+) T lymphocytes is specific to leukemia, we have sought to extend the scope of our hypothesis on the antitumor role of the innate CD8(+) T lymphocytes by assessing the presence of these cells in solid tumors or metastasized tissues. The characteristics of tumor micro-environments differ according to cancers, and it is for that reason that we have assessed the presence of innate CD8(+) T lymphocytes in two types of solid tumors: breast cancer and ovarian cancer.

**Figure 6:**
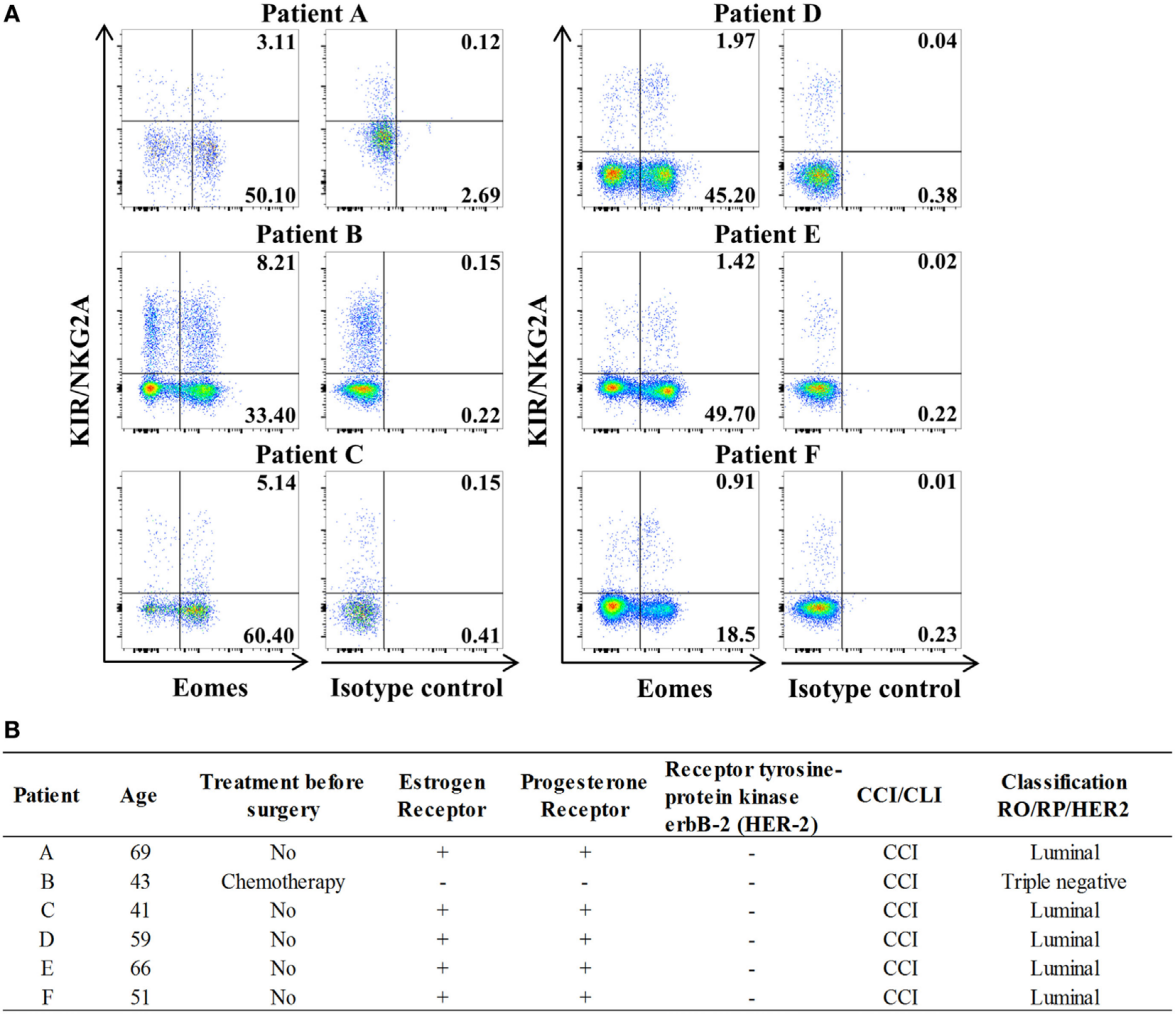
**Innate CD8(+) T cells are present in tumor-draining lymph nodes (TDLNs) from breast cancer patients**. **(A)** Frequencies of innate CD8(+) T cells among total CD8(+) T cells in axillary TDLNs were analyzed *ex vivo* by flow cytometry. The full cohort of five luminal breast cancer patients and one triple negative patient with chemotherapy is shown. Quadrant frequencies are displayed in upper right corners. **(B)** Clinical patient characteristics. CCI: invasive ductal carcinoma. CLI: invasive lobular carcinoma.

Study of lymph nodes (LN) invaded by tumor cells in breast cancer has highlighted the significantly frequent presence of innate CD8(+) T lymphocytes (Figure 6). The results of this preliminary study seem to justify organization of a large-scale study on breast cancer patients aimed at determining a possible link between, on the one hand, the numerical and functional status of the innate CD8(+) T cells present in the invaded LN draining the tumor and, on the other hand, disease prognosis.

**Figure 7:**
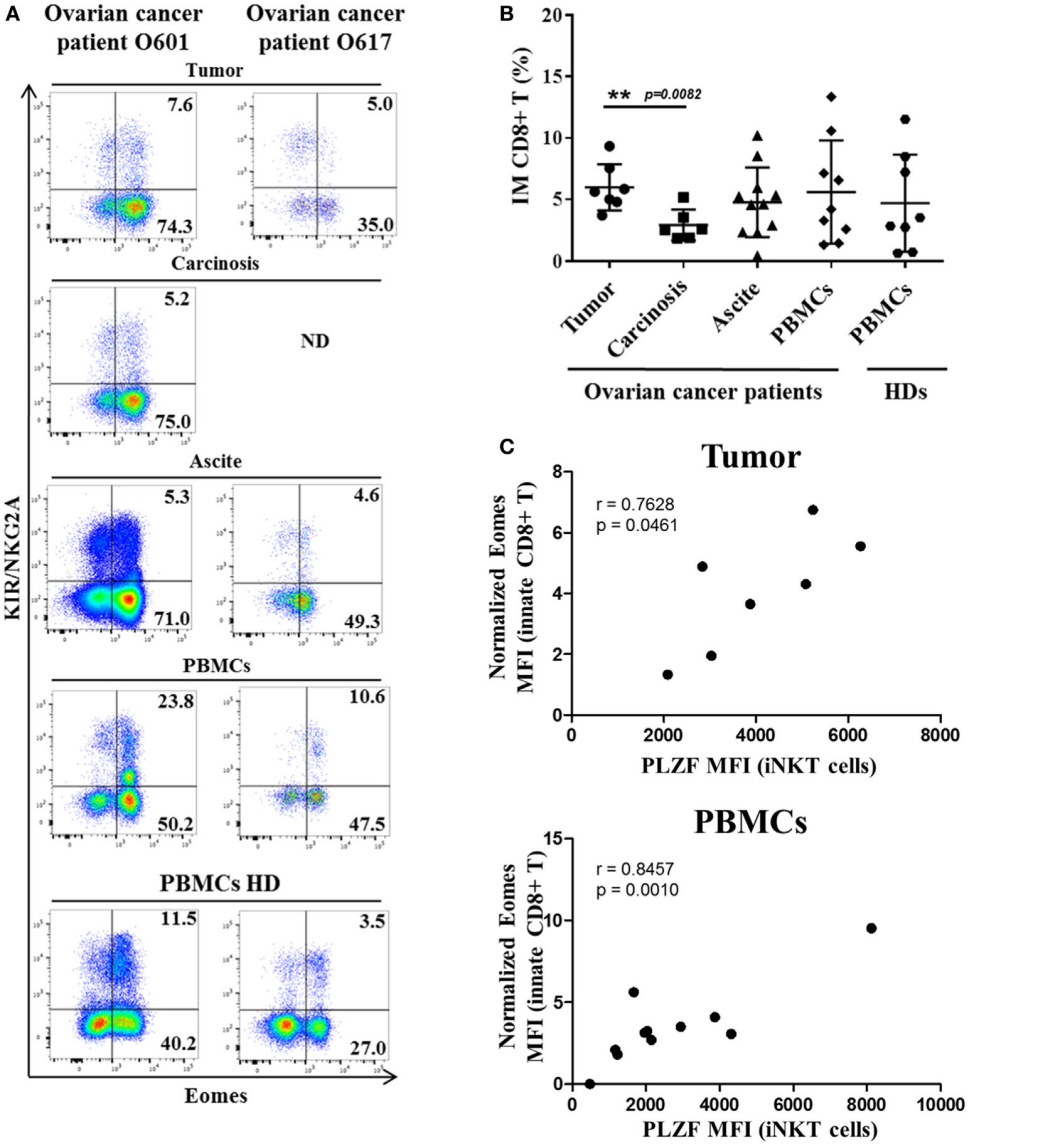

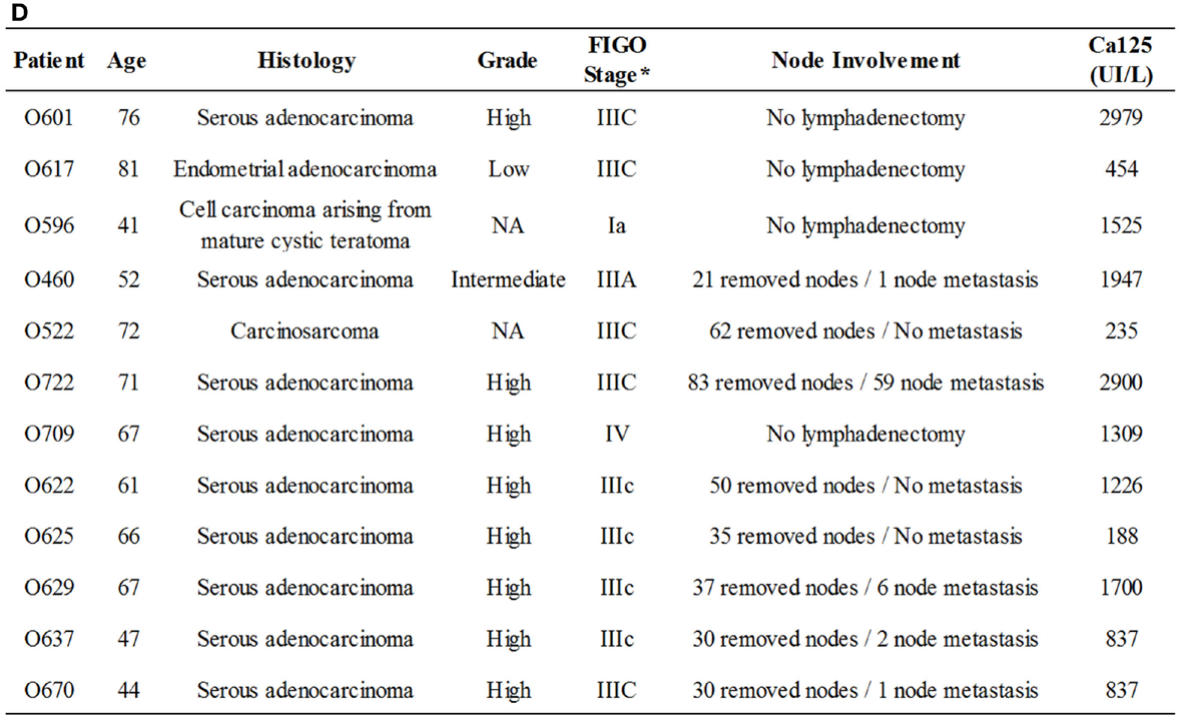
**Innate CD8(+) T cells are present in tumors and tumor fluids from ovarian cancer patients**. Cells from tumors, carcinoma, peritoneal ascites, and peripheral blood mononuclear cells (PBMCs) from ovarian cancer patients and PBMCs from healthy individuals [healthy donors (HDs)] were analyzed *ex vivo* by flow cytometry. **(A)** Two representative samples reflecting the presence of innate CD8(+) T cells in tumor, carcinoma, peritoneal ascites, and PBMCs from ovarian cancer patients and PBMCs from two HDs. ND, not determined. **(B)** Cohort study of innate CD8(+) T-cell frequency [expressed as percentage ± SD of T cells among total CD8(+) T cells] in tumors (*n* = 7), carcinoma (*n* = 7), peritoneal fluids (*n* = 12), and PBMCs from ovarian cancer patients (*n* = 10) and PBMCs from HDs (*n* = 8). **(C)** Eomes and promyelocytic leukemia zinc-finger factor (PLZF) expression were analyzed in innate CD8(+) T cells [CD3(+) CD8(+) KIR/NKG2A(+) Eomes(+) cells] and iNKT cells [CD3(+) 6B11(+) cells], respectively. Eomes MFI values are expressed relative to that of CD45(+) CD3(−) KIR/NKG2A(−) cells. The MFI of PLZF-expressing iNKT cells correlate positively with Eomes MFI in innate CD8(+) T both in tumor and PBMCs but not in carcinosis (*r* = −0.04673, *p* = 0.8853) and ascites (*r* = 0.5424, *p* = 0.2084) from ovarian cancer patients (correlation Spearman test). **(D)** Clinical patient characteristics. NA, not available.

Study of intra-tumoral lymphocytes in ovarian cancer has shown the significantly frequent presence of innate CD8(+) T lymphocytes, which are also present in the peritoneal carcinosis of ovarian cancers and in ascite fluids (Figure 7). Interestingly, there was a significant higher frequency of innate CD8(+) T lymphocytes in primitive tumors than in carcinosis (a proximity metastasis), indicating that these cells not only penetrate tumors in ovarian cancer but also might undergo immune subversion in the peritoneal environment. Moreover, like in CML, our data have shown a positive correlation between Eomes expression in innate CD8(+) T lymphocytes and PLZF expression in iNKT cells both in peripheral blood mononuclear cells (PBMCs) and tumor material (but not in carcinosis and ascite) from ovarian cancer patients (Figure 7C).

Taken as a whole, these different results show that innate CD8(+) T lymphocytes are present in tumors and probably integrated in the dynamics of anticancer responses. However, at this time, it is not possible to determine their intra-tumoral functional status and, more specifically, their prognostic usefulness.

### Innate CD8(+) T Lymphocytes and Immune Exhaustion in Cancer Patients

Numerous studies have dealt with the role of Eomes in the exhaustion of CD8(+) T lymphocytes during chronic infections or cancers (68–72). Our results in healthy donors (HDs) attesting a significant proliferative response to IL-15 (Figure S4 in Supplementary Material) do not lend support to an exhaustion phenotype of innate CD8(+) T cells in normal conditions. During cancer progression, on the other hand, the elevated frequency of innate CD8(+) T lymphocytes in contact with the tumor present in severely ill patients raises the question of their possible exhausted immune-exhaustion status. Furthermore, during CML, we have observed a functional defect in the innate CD8(+) T-cell population, a finding suggesting not only that CML innate CD8(+) T cells are in the process of being exhausted but also that they could be a target of immune blocking checkpoints. Monitoring multifunctionality of immune-exhausted CD8(+) T cells co-expressing Eomes and KIR/NKG2A in patients with solid tumors and CML will help to determine whether the evasion/subversion mechanisms used by tumor cells also apply to innate CD8(+) T cells in humans. At this stage, it would be interesting to determine whether the innate CD8(+) T cells express CXCR3 and CXCR5, two chemokine receptors that were recently described as being associated with CD8(+) T lymphocytes during cancers or chronic viral infection and of which the proliferation is restored following anti-programmed cell death 1 treatment (73).

## Conclusion

Taken together, having availed ourselves of different types of empirical data, we propose that in humans, innate CD8(+) T lymphocytes constitute a new cellular component that could have a role in antitumor immunity. Our results during CML and ovarian cancer are in favor of the existence of an axis composed of innate T cells with an antitumoral potential, which consist of iNKT cells and innate CD8(+) T lymphocytes. Differentiation of these unconventional CD8(+) T cells in humans is associated with Eomes expression and could depend on IL-4 and PLZF(+) iNKT cells. Our results suggest that during CML, these innate CD8(+) T lymphocytes could be controlled by immune checkpoints. While the results of our studies on solid cancers corroborate our hypothesis concerning the role of innate CD8(+) T lymphocytes in antitumor immunity, as of now we are unable to determine whether this role is protective, permissive, and/or detrimental in these other types of cancer.

## Methods

### PBMCs from HD

Healthy donors were volunteers from the Pôle Biologie Santé (Poitiers, France). PBMCs were isolated from blood samples by density gradient centrifugation (Histopaque^®^-1077, Sigma-Aldrich), resuspended in 90% fetal calf serum with 10% DMSO, and placed in a controlled rate freezer for cryopreservation at −80°C until use. For this series of data, age range was between 21 and 65 with a sex ratio of 0.6.

### Clinical Breast and Ovarian Cancer Samples

Invaded tumor-draining lymph nodes were collected from six untreated luminal breast cancer patients undergoing standard surgery at Institute Curie Hospital (Paris, France), in accordance with institutional ethical guidelines. Precisely, all patients gave informed consent in a written form in accordance with the Declaration of Helsinki for participation in this study, which was approved by the scientific committee of the Institute Curie Hospital.

Cells from tumors, carcinomatosis, peritoneal ascites, and PBMCs were collected from eight untreated patients with ovarian carcinoma undergoing standard surgery at CHU of Rennes. Human samples were obtained from the processing of biological samples through the Centre de Resources Biologiques Santé of Rennes (BB-0033-00056). The research protocol was conducted under French legal guidelines and fulfilled the requirements of the local institutional ethics committee.

Tissue samples were cut into small fragments, digested with 0.1 mg/ml Liberase TL (Roche) in the presence of 0.1 mg/ml DNase (Roche) for 15–30 min before the addition of 20% FCS. Cells were filtered on a 40-μm cell strainer (BD), washed, and cryopreserved for further study. Ascite cells were obtained after centrifugation (400 *g*, 10 min).

### Experimental Studies in Animals

The 8-to-12-week-old female C57BL/6JRj Eomes-GFP transgenic mice (74) and BALB/c wild-type mice (Janvier) were used and bred in our animal facility (PREBIOS, Platform of Research and Experimentation in Health Biology of the University of Poitiers) under specific pathogen-free conditions. Spleen and thymus were collected immediately after cervical dislocation. Splenocytes and thymocytes were isolated and analyzed *ex vivo* by flow cytometry. In some experiments, splenocytes were cultured for 16 h in the presence of 20 ng/ml of each cytokine (IL-12: R&D Systems; IL-18: MBL International) prior to analysis by flow cytometry. All procedures were performed in accordance with the recommendations of the European Accreditation of Laboratory Animal Care and French institutional committee of Poitou-Charentes (COMETHEA, C2EA-84, no. 2016072216352833).

### Cell Culture and Functional Assays

Peripheral blood mononuclear cells (1 × 10^6^ cells/ml) were cultured in RPMI 1640 medium supplemented with 10% heat-inactivated FCS and antibiotics. For IL-12 + IL-18 or IL-15 stimulation, PBMCs from HD were seeded at 1.10^6^ cells/ml into 24-well plates and incubated for 48 h with 20 ng/ml of each cytokine (IL-12: R&D Systems; IL-18: MBL International; IL-15: Miltenyi). Golgistop (BD Biosciences) was added for the last 5 h of culture for IL-12 + IL-18 stimulation.

### Flow Cytometry

In humans, phenotypic analysis of cells from HD or breast/ovarian cancer patients was performed by flow cytometry either *ex vivo* or after culture. Expression of different markers was assessed by staining with appropriate combinations of the following antibodies (mAbs): anti-TCR-αβ BV421 (clone: IP26, BioLegend), anti-CD8 PE-Cy7 (clone: RPA-T8, Biolegend), anti-IFN-γ FITC (clone: B27, BioLegend), anti-TCR-Vα7.2 BV421 (clone: 3C10, BioLegend), anti-CD161 PerCP-Cy5.5 (clone: HP-3G10, Biolegend), and anti-Eomes eFluor^®^ 660 (clone: WD1928, eBiosciences). KIR/NKG2A referred to staining with the mix of the three following antibodies from Miltenyi Biotech: anti-KIR2D PE (clone: NKVFS1), anti-KIR3DL1/KIR3DL2 (CD158e/k) PE (clone: 5.133), and anti-NKG2A (CD159a) PE (clone: REA110). For nuclear Eomes staining and intracytoplasmic IFN-γ staining, cells were permeabilized with an anti-human FoxP3 staining kit (eBioscience) and a Cytofix/Cytoperm kit (BD Biosciences), respectively.

In mice, splenocytes and thymocytes were stained with appropriate combinations of the following antibodies: anti-TCR-β PerCP-Cy5.5 (clone: H57-597, BD Biosciences), anti-CD8 BV510 (clone: 53-6.7, BD Biosciences), anti-CD44 PE-Cy7 (clone: IM7, BD Biosciences), anti-CD49d Vioblue (clone: R1-2, Miltenyi Biotec), anti-CD122 APC (clone: TM-B1, Biolegend), anti-CD4 PE (clone: RM4-5, BD Biosciences), anti-CD24 PE (clone: M1/69, BD Biosciences), and anti-Eomes AF488 (clone: Dan11mag, eBioscience). For nuclear Eomes staining and intracytoplasmic IFN-γ staining, cells were permeabilized with an anti-human FoxP3 staining kit (eBioscience).

Dead cells were excluded using the Live/Dead^®^ Fixable NearIR Dead Cell Stain kit (Life Technologies). Cells were analyzed on a Fortessa flow cytometer (BD Biosciences) or a FACSVerse™ cytometer with FACSuite™ software (BD Biosciences) using FlowJo v10 (TreeStar, Inc.).

### Statistical Analysis

Statistical analyses were performed using GraphPad Prism version 7.0 (GraphPad Software). The statistical significance of differences and of mean values was analyzed by the two-tailed Wilcoxon test in Figure 3, paired *t*-test in Figure 4, and Mann–Whitney non-parametric test in Figure 7. Results were considered to be statistically significant when *p* < 0.05.

## Ethics Statement

All patients gave informed consent in a written form in accordance with the recommendations of “the Declaration of Helsinki” for participation in the study, which was approved by the scientific committees of the Clinic Investigator Center Inserm CIC-1402 (Poitiers, France), the Biological Resource Center of CHU of Poitiers (NF S96-900 certification since February 2014), the Biological Resource Center of CHU of Rennes (NF S96-900, certification since May 2009), and the Institute Curie Hospital (Paris, France).

## Author Contributions

AB, EC, and FJ designed the experiments, performed the experiments, analyzed and interpreted the data, and wrote the manuscript. LL, MA, NN, NP, BM, and SB contributed to sample preparation from patients and healthy controls, designed the experiments, performed the experiments, and analyzed and interpreted the data. EP, VC, and VL provided clinical samples and contributed to the interpretation of data. AH and J-MG together were responsible for the overall study design, supervised the project, and took primary responsibility for writing the manuscript.

## Conflict of Interest Statement

The authors declare that the research was conducted in the absence of any commercial or financial relationships that could be construed as a potential conflict of interest.
