# Supplementary figures and images for: Phenotype of NK-Like CD8(+) T Cells with Innate Features in Humans and Their Relevance in Cancer Diseases
Source: Front Immunol. 2017 Mar 27;8:316. doi: 10.3389/fimmu.2017.00316 (PMC5366313; doi:10.3389/fimmu.2017.00316)

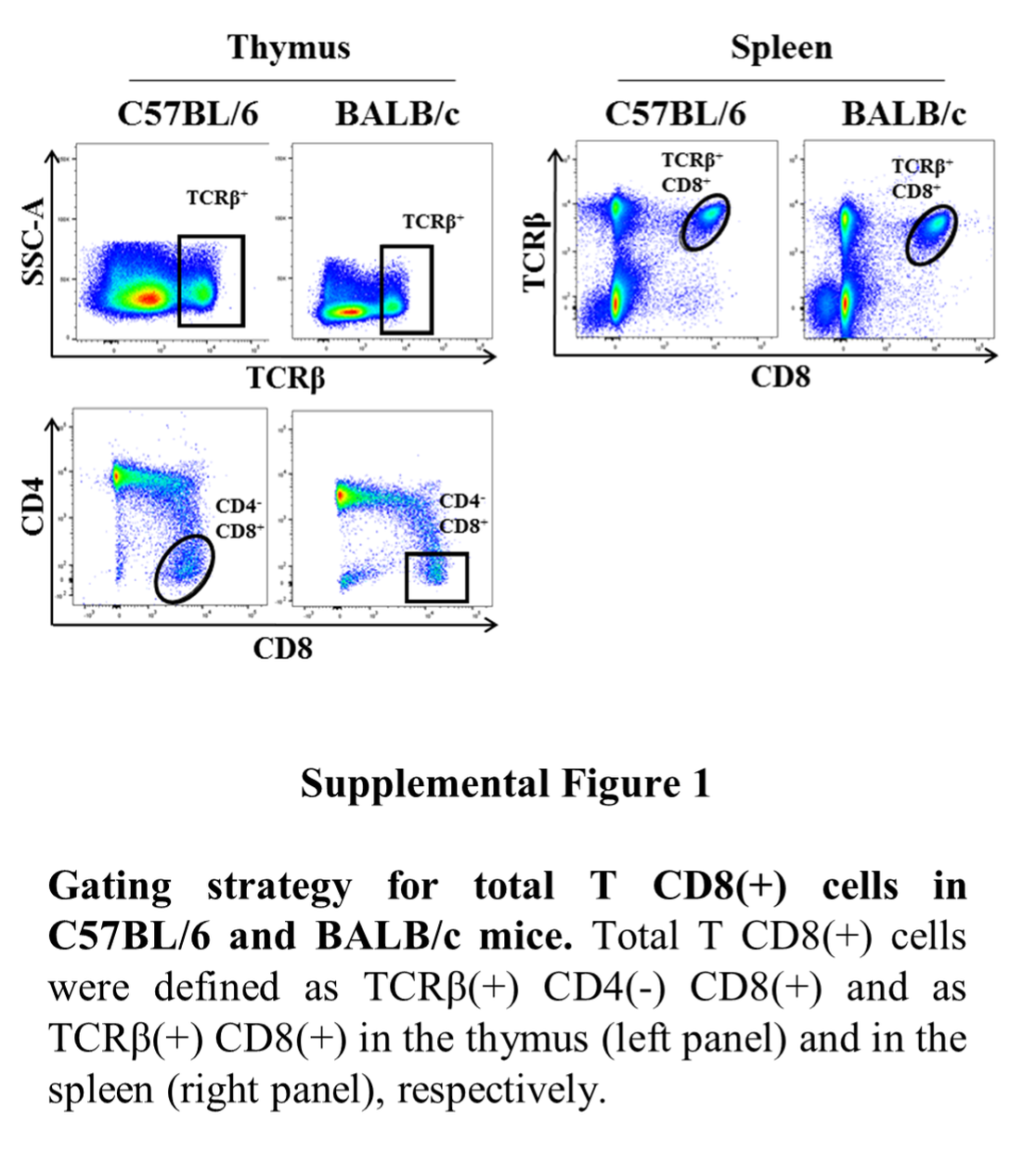

Supplement: Supplementary file 1 [file Image_1.TIF]

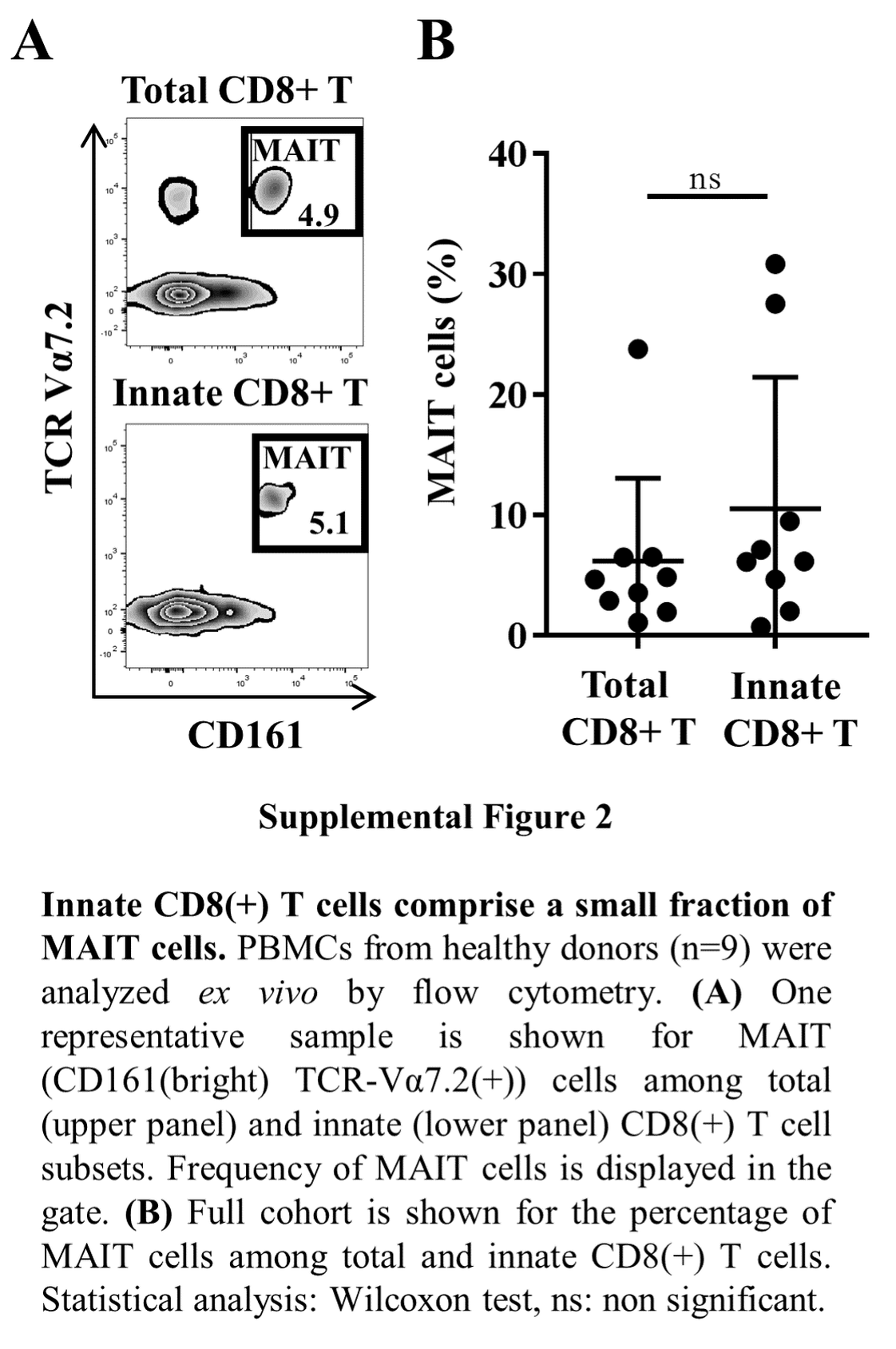

Supplement: Supplementary file 2 [file Image_2.TIF]

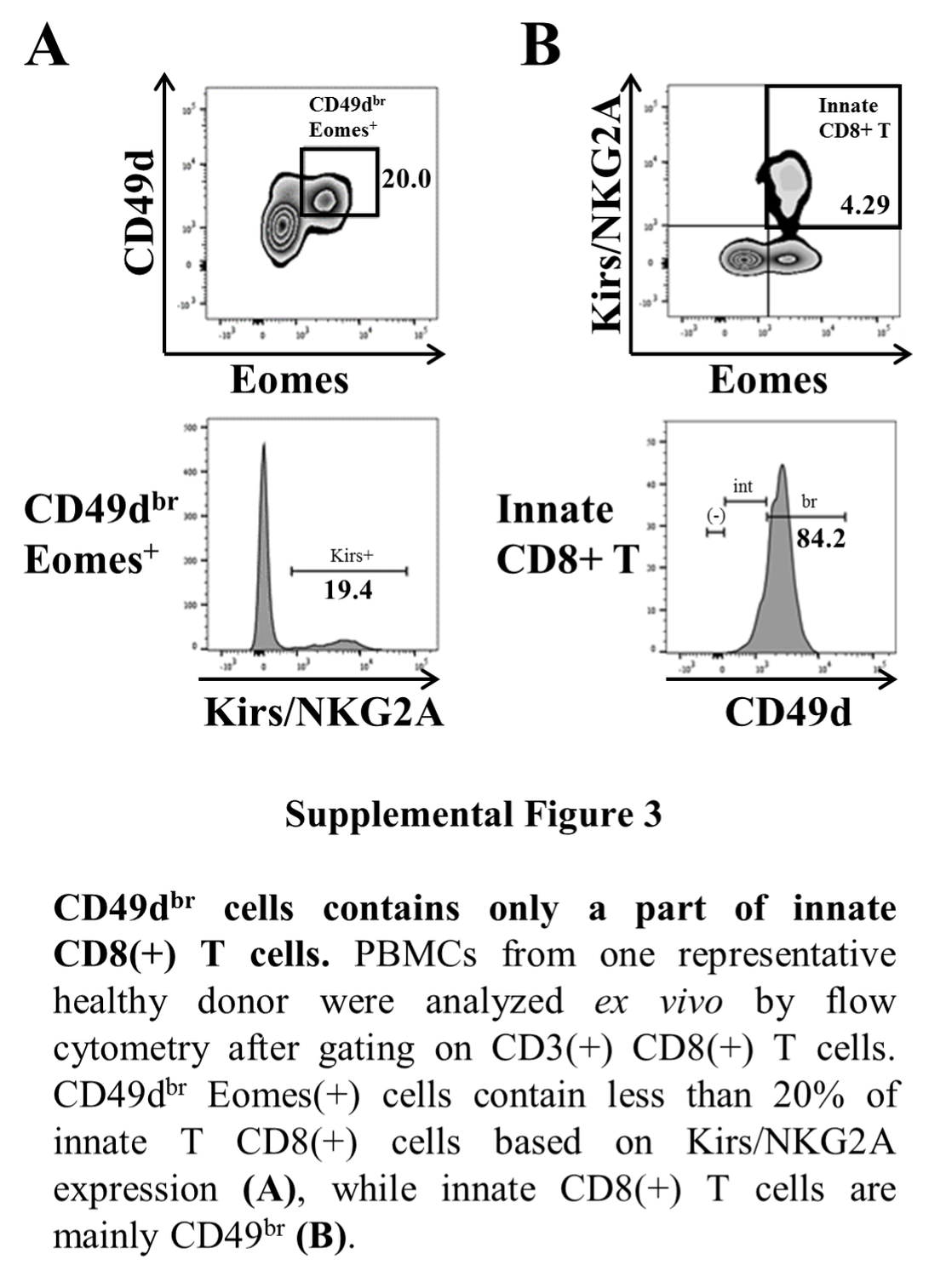

Supplement: Supplementary file 3 [file Image_3.TIF]

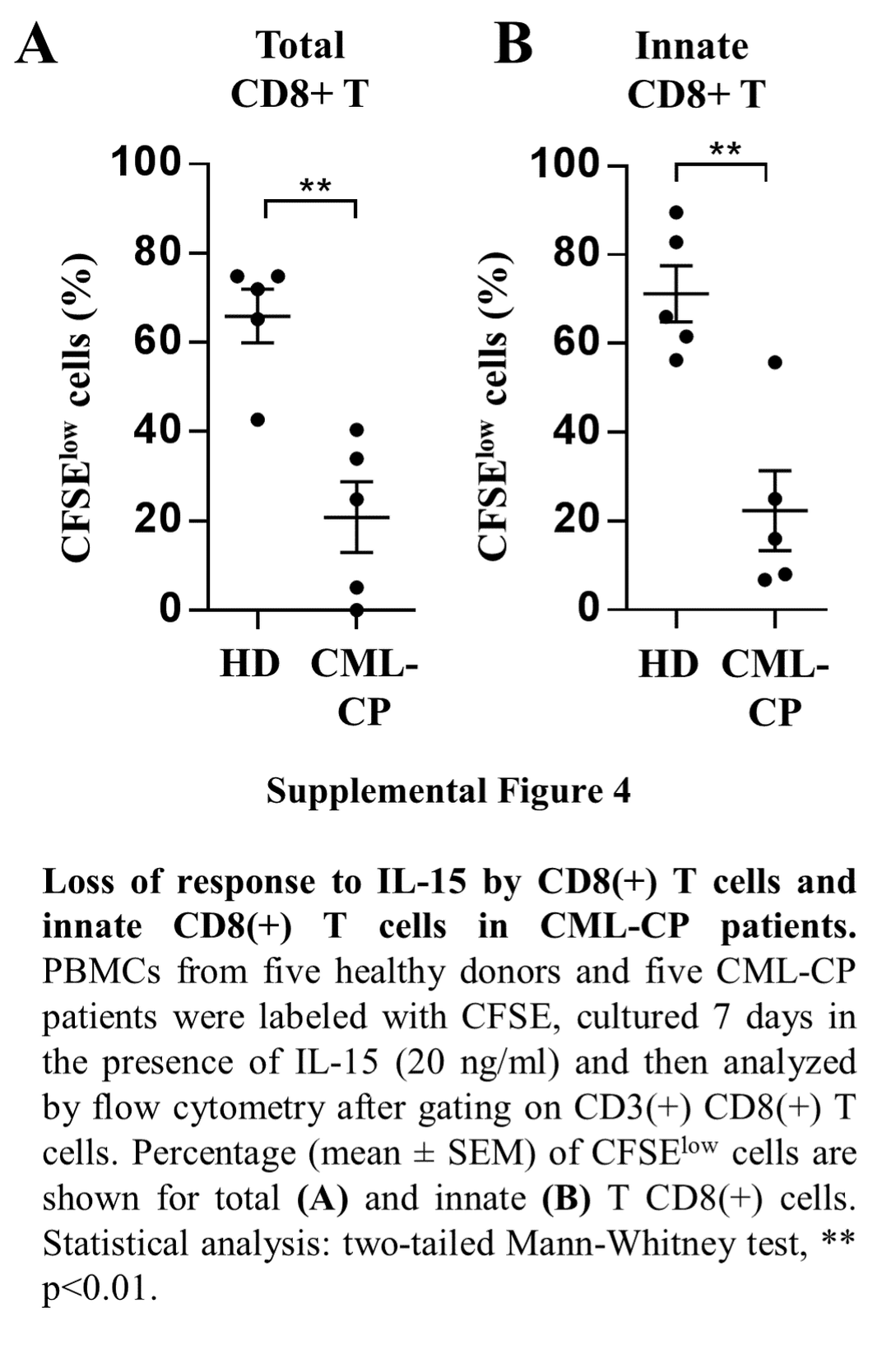

Supplement: Supplementary file 4 [file Image_4.TIF]
